# Aortic shelf as a normal variant diagnosed primarily as the aortic dissection: A case report

**DOI:** 10.34172/jcvtr.2020.41

**Published:** 2020-07-19

**Authors:** Mehrnoush Toufan Tabrizi, Naser Khezerlouy-Aghdam, Venus Shahabi Raberi, Ahmad Jamei Khosroshahi

**Affiliations:** ^1^Cardiovascular Research Center, Tabriz University of Medical Sciences, Tabriz, Iran; ^2^Department of Cardiology, Urmia University of Medical Sciences, Urmia, Iran

**Keywords:** Aortic Shelf, Traumatic Aortic Dissection, Descending Thoracic Aorta, Transesophageal Echocardiography

## Abstract

Traumatic aortic dissection is most commonly caused following sudden deceleration injury. It most commonly involves descending thoracic aorta (DTA) and is associated with high mortality and morbidity if not treated urgently. Confirmation of diagnosis often requires contrast-enhanced computed tomography (CECT) or magnetic resonance imaging (MRI), which is time consuming, expensive, and often not available at many health-care facility. Transesophageal echocardiography (TEE) is equally efficient to CECT and MRI in diagnosing aortic dissection. It may also provide additional information that can be very useful for the management of the patient. In some cases, the likelihood of error in the diagnosis of such a critical condition with normal cardiovascular variations is expected. Herein, we describe a case with primary diagnosis of aortic dissection that led to final diagnosis of an aortic shelf that medically managed with a good long-term prognosis. In patients suspected to aortic dissection due to any cause, the specialized evaluation using the most accurate and sensitive tools is strongly recommended to discriminate normal vascular variations from major vascular defects requiring emergent surgical interventions.

## Introduction

Traumatic aortic dissection is most commonly caused following sudden deceleration injury. It most commonly involves descending thoracic aorta (DTA) and is associated with high mortality and morbidity if not treated urgently. Confirmation of diagnosis often requires contrast-enhanced computed tomography (CECT) or magnetic resonance imaging (MRI), which is time consuming, expensive, and often not available at many health-care facility. Transesophageal echocardiography (TEE) is equally efficient to CECT and MRI in diagnosing aortic dissection. It may also provide additional information that can be very useful for the management of the patient.^1^ Understanding the progression and severity of this condition is very important to choose the best early treatment approach. Several diagnostic tools have been recently developed to early detection of aortic dissection including ultrasonography, computed tomography, and magnetic resonance imaging.^2^ However, despite high precision of such diagnostic tools to detect aortic dissection, in some cases, the likelihood of error in the diagnosis of such critical condition with other cardiovascular benign or even normal variations that requires only medication or elective managements, not surgical endovascular interventions is expected.^3^ One of these variations is aortic shelf as a rare congenital variation in aorta that placed in differential diagnosis with aortic dissection, but with a completely different prognosis as well as different treatment approach.^4^ Herein, we describe a case with primary diagnosis of aortic dissection that led to final diagnosis of an aortic shelf that medically managed and led to a good long-term prognosis.

## Case Presentation

**Figure 1:**
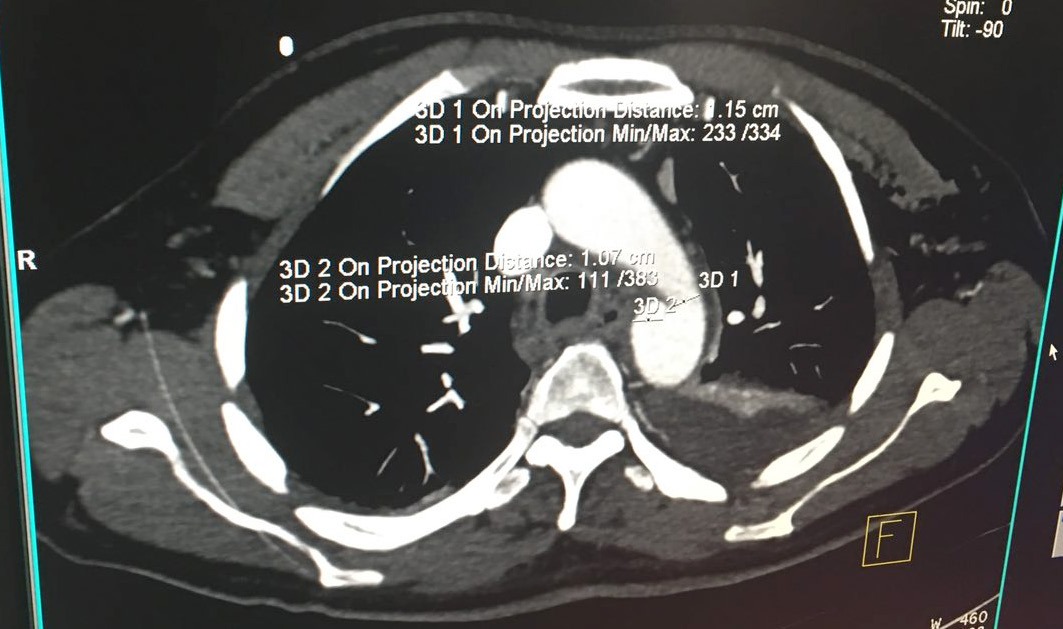
CT imaging feature, a hypo-dense area at proximal part of descending aorta with an enfolding suggesting for peri-aortic hematoma and a possible aortic dissection

**Figure 2:**
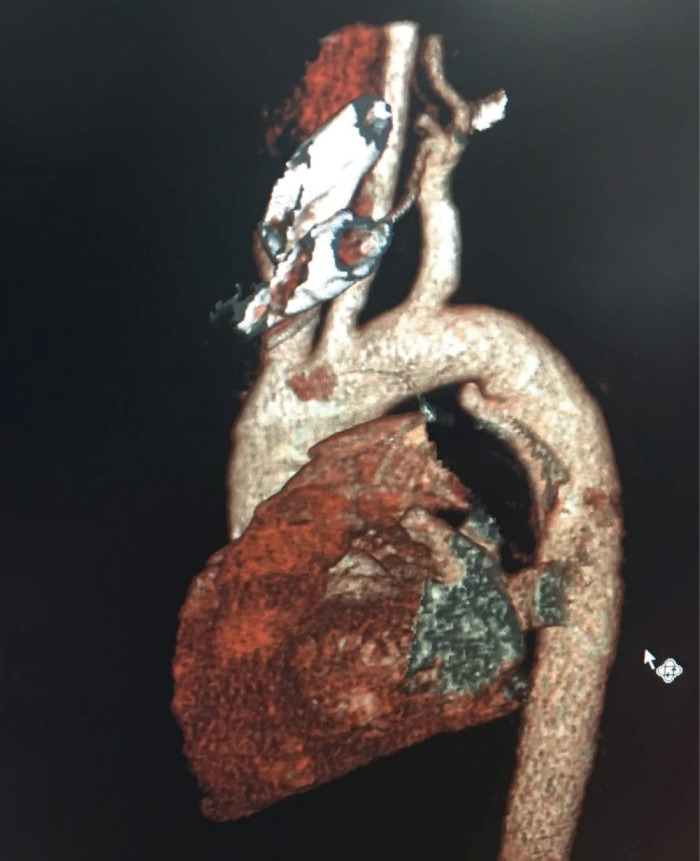
CT angiography feature with localized dilatation of proximal descending aorta at medial aspect (arrow shows the site of dilatation)

**Figure 3:**
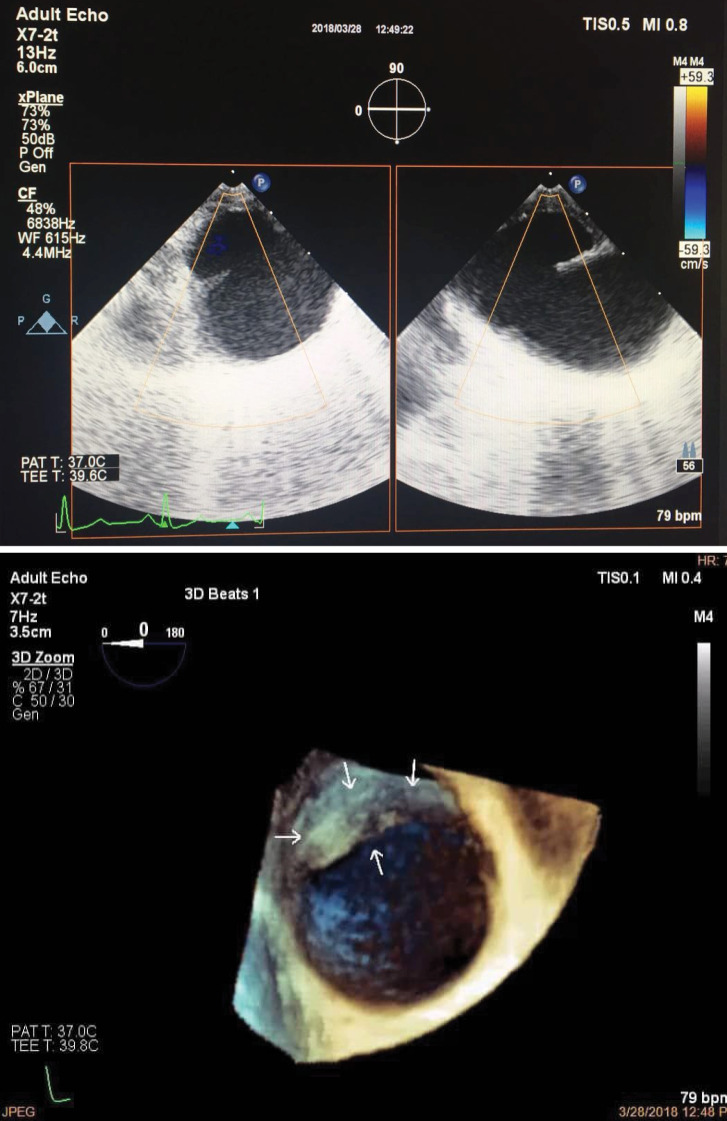
(A) A localized dense shelf-like enfolding perpendicular to the aorta as a normal variant was visible without any evidence of hematoma, turbulent flow, typical flap, or false lumen without any evidence of aortic dissection. (B) A localized dense shelf-like enfolding perpendicular to the aorta as a normal variant was visible without any evidence of hematoma, turbulent flow, typical flap, or false lumen without any evidence of aortic dissection

A 50-year smoker man who referred to emergency department due to multiple trauma caused by car accident was initially managed. In chest X-ray, fracture in the posterior arch of left side seventh to ninth chest ribs , bilateral pleural effusion and left side pneumothorax were revealed that managed by left side chest tube insertion. Bed side FAST was also considered detecting any abnormal finding. In laboratory test, there was no evidence in decreased hemoglobin level. The patient had a stable hemodynamic status. Chest CT scan revealed a periaortic hypo-dense area proximal to descending aorta suggesting hematoma and also a possible feature of dissection flap suggesting a traumatic descending aortic dissection. In emergent transthoracic echocardiography on the first day of admission, a localized dilatation with a dimension (16*18 mm) in medial side of proximal descending aorta was found, but there was not turbulent flow or evident FLAP attributable to aortic dissection. In CT angiography of the aorta (Figure 1), a vertical flap (length =12 mm) in medial side of proximal descending aorta just after arch was seen suggestive for dissection. According to CT angiography findings (Figure 2), the patient was candidate for elective thoracic endovascular repair (TEVAR); but in transesophageal echocardiography, a localized dense shelf-like enfolding perpendicular to the aorta at medial side as a normal variant was visible without any evidence of hematoma, turbulent flow, typical flap or false lumen and any other evidence of aortic dissection (Figure 3). Also patient had left sided pleural effusion and lung atelectasis. Thus, the patient was referred to cardiology department for further evaluation. Follow up the patient over the next few days showed no evidence of hemodynamic instability, decrease in blood pressure, or serum hemoglobin decline. Within hospitalization, the chest pain gradually reduced. In esophageal echocardiography two weeks and one month later did not show any new changes and the previous diagnosis (aortic shelf) was confirmed. Finally the patient was managed medically with control echocardiography at follow up. In the six-month follow up, the patient had good clinical condition without any complication.

## Discussion

Unfortunately, the presence of natural aortic shelf as a normal variation may be misleading to professionals interpreting it as a life-threatening condition such as aortic dissection. This misdiagnosis may be even to the wrong treatment approach. In this regard, using a combination of imaging modalities to achieve a true definitive diagnosis (differentiation of normal variants from life-threatening conditions needing urgent interventional approaches) is advisable.

In the region of the ductus arteriosus an anterior or medial shelf may normally exist just proximal to the aortic origin of the ductus. It is important not to mistake this for a typical posterior coarctation shelf. In addition, the size of the transverse arch must also be noted as it invariably is somewhat small, particularly in the newborn with severe coarctation.^5,6^ But in some cases, aortic shelf may be an evidence of cardiovascular anomalies needing repairing interventions early in life. For instance, imaging the coarctation of the aorta may be led to appearing a posterior aortic shelf commonly in fetus but rarely in adulthood.^7^ In other word, the coarctation of the aorta in adults may appear as a localized posterior shelf-like thickening of the aortic wall. Although, most of these abnormalities are diagnosed as vascular anomalies needing surgical repairing approaches, in most cases, vascular shelf especially in aorta may be considered only as a normal variation without any evidence of hemodynamic abnormalities or further life-threatening events. In the case presented here, the diagnosed was primarily focused aortic dissection due to precedent trauma but the absence of turbulent flow or evident FLAP strongly raised the suspicion of this diagnosis. In this regard and by using more accurate diagnostic modality including CT angiography and esophageal echocardiography, the diagnosis of aortic dissection was completely ruled out. Thus, the patient was finally monitored and followed-up for a six-month period showing reducing clinical manifestations without any long-term complications. So in patients suspected to have localized aortic dissection at proximal DAO due to any cause without strong clinical evidences, the specialized evaluations using the most accurate and sensitive tools is strongly recommended to discriminate normal vascular variations from major vascular defects requiring emergent surgical interventions.

## Competing interests

None declared.

## Funding

None.
